# Laser cutting machine-induced maculopathy and spontaneous recovery

**DOI:** 10.5935/0004-2749.20220014

**Published:** 2022

**Authors:** Selim Cevher

**Affiliations:** 1 Hıtıt Unıversıty Medıcıne Faculty, Department of Ophthalmology, 019030 Corum, Turkey

**Keywords:** Maculopathy, Macular degeneration, Tomography, optical coherence, Laser, Maculopatia, Degeneração macular, Tomografia de coerência óptica, Laser

## Abstract

The thermal effects of laser cutting machines could damage the macula. A few studies in the literature have described macular injury induced by industrial laser burns. The aim of this study was to report the clinical, visual, and optical coherence tomography findings in a gold refinery worker with laser-induced maculopathy. A 21-year-old male gold refinery worker had vision loss in his right eye after using a laser cutting machine without wearing laser eye protection gear. At the first visit (24 h later), his best-corrected visual acuity was 7/10 in the right eye and 10/10 in the left eye. The anterior segment examination was normal. In fundus examination, focal, round, and yellowish lesion was detected within the fovea. The optical coherence tomography findings were foveal outer retinal disruptions and irregularities extending from the outer plexiform layer to the retina pigment epithelium. After 4 months, the best-corrected visual acuity had improved to 1.0, and the optical coherence tomography findings had resolved.

## Abstract (pt)

Os efeitos térmicos das máquinas de corte a laser podem danificar a mácula. Poucos estudos na literatura tem descrito a lesão macular por queimadura a laser industrial. Neste estudo, objetivamos relatar os achados clínicos, visuais e de tomografia de coerência óptica em um trabalhador de refinaria de ouro com maculopatia induzida por laser. O trabalhador da refinaria de ouro de 21 anos teve perda de visão no olho direito depois de usar uma máquina de corte a laser sem usar equipamento protetor ocular para laser. Sua acuidade visual melhor corrigida foi de 7/10 no olho direito e 10/10 no olho esquerdo na primeira consulta (24h depois). O exame do segmento anterior estava normal. No exame de fundo de olho, lesões focais, redondas e amareladas foram detectadas dentro da fóvea. Os achados da tomografia de coerência óptica revelaram rupturas na fóvea da retina externa e irregularidades estendendo-se da camada plexiforme externa ao epitélio pigmentar da retina. Após 4 meses, a acuidade visual melhor corrigida melhorou para 1,0 e os achados da tomografia de coerência óptica foram revigorados.

## INTRODUCTION

Retina pigment epithelium (RPE) and choroid absorb the light at a certain capacity and protect the retina from the phototoxic and thermal effects of light. However, retinal damage may occur if the effects of light exceed the capacity for protection. Some factors, such as duration of exposure, power of the device, wavelength, and degree of retinal pigmentation, are important for macular damage^(1)^.

Laser-induced maculopathies have been reported by several researchers in the literature. Numerous of these cases were associated with use of a laser pointer device. Laser machines are used in various occupations, such as in a gold refinery. Macular injury may occur when workers use such devices (e.g., a laser cutting machine) without wearing any protective equipment.

In this study, we report a case of laser-induced maculopathy caused by a laser cutting machine, and the spontaneous improvement of clinical, visual, visual field, and optical coherence tomography (OCT) findings.

## CASE REPORT

**Figure 1:**
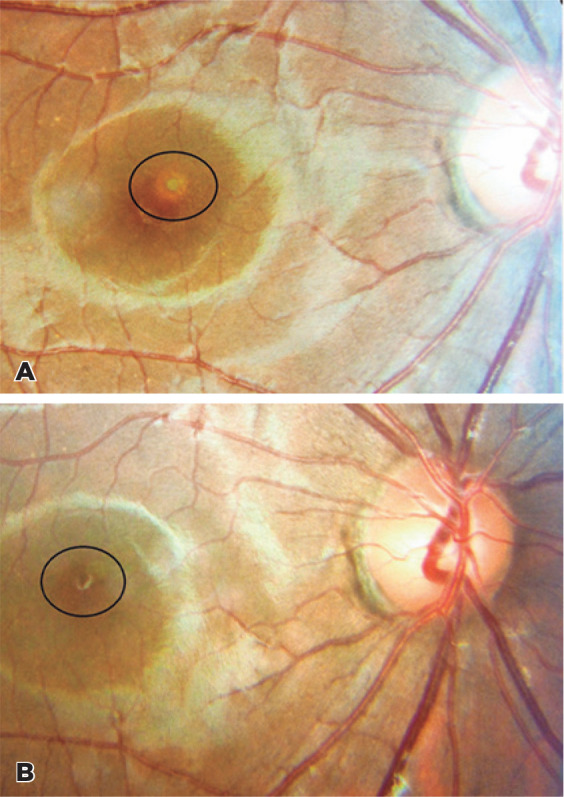
A) Focal, round, and yellowish lesion within the fovea. B) The foveal yellowish lesion after 4 months (smaller than the first visit).

**Figure 2:**
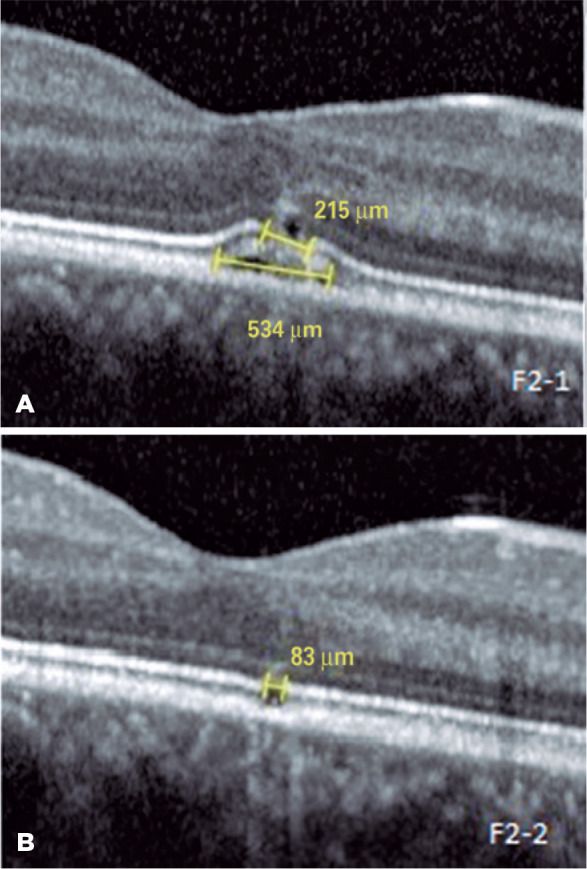
A) Foveal outer retinal disruptions and irregularities extending from the external limiting membrane to the retina pigment epithelium in OCT. The size of the ellipsoid zone damage and RPE damage was 215 _µ_m and 534 _µ_m, respectively. B) After 4 months, minimal defect was observed on the ellipsoid zone and RPE (83 _µ_m).

**Figure 3:**
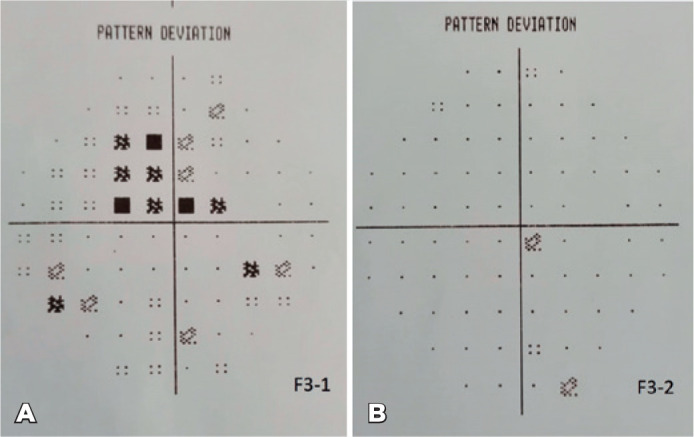
A) Small central scotoma in the computerized 10-2 visual field threshold test. B) After 4 months, the small central scotoma in the computerized 10-2 visual field threshold test had disappeared. 90 Arq Bras Oftalmol. 2022;85(1):88-91

A 21-year-old patient presented to our clinic with visual loss and central scotoma in his right eye, which developed acutely after exposure to laser light from a laser cutting machine (1,060 nm, 70 W) for a few seconds while working in a gold refinery. Although he closed his left eye while working without wearing laser eye protection gear, the laser light suddenly reflected on his right eye. His best-corrected visual acuity (BCVA) on the Snellen chart was 7/10 in his right eye and 10/10 in his left eye. The anterior segment examination was normal. Intraocular pressure (Goldman applanation) was 15 mmHg in the right eye and 13 mmHg in the left eye. In fundus examination, focal, round, and yellowish lesion was detected in the fovea (Figure 1A). Foveal outer retinal disruptions and irregularities extending from the external limiting membrane to the retina pigment epithelium were detected using OCT (Spectralis SD-OCT; Heidelberg Engineering, Heidelberg, Germany) (Figure 2A). The size of the ellipsoid zone damage and RPE damage was 215 µm and 534 µm, respectively. A computerized 10-2 visual field threshold test (Humphrey Automated Perimeter; Humphrey Instruments, San Leandro, CA, USA) showed small central scotoma in the right eye and a normal field in the left eye (Figure 3A). The hole-in-the-card test, used for the detection of the dominant eye, was utilized to explain the reasons responsible for the occurrence of damage only in the right eye. Testing revealed that the right eye was the dominant eye. Finally, laser-induced photic maculopathy was diagnosed in the right eye, and no treatment was recommended. Follow-up was decided. After 4 months, BCVA improved to 10/10 and the foveal yellowish lesion became smaller (Figure 1B). The OCT findings recovered with a minimal defect on the ellipsoid zone and RPE (83 µm) (Figure 2B). The defect size improved from 215 µm to 83 µm. The visual field analysis was normal (Figure 3B).

OCT= optical coherence tomography; RPE= retina pigment epithelium.

## DISCUSSION

Accidental laser-associated maculopathy occurs infrequently. The effects of laser on the retina include outer retinal disruption, foveal hemorrhage, macular edema, epiretinal membrane, macular hole, and choroidal neovascularization^(2)^. The Food and Drug Administration recognizes four major hazard classes (I to IV) of lasers, including three subclasses (IIa, IIIa, and IIIb). Higher classes indicate more powerful lasers and greater potential for serious injury if used improperly.

In both solar and laser-induced retinopathies, the primarily affected structures are the foveal photoreceptor outer segments and RPE^(3)^. Initially, a yellowish lesion is observed in the fovea, which appears to correspond to changes in the hyperreflective photoreceptor bands on OCT^(4)^. Subsequently, the yellowish lesion may transform into a reddish cyst-like lesion with surrounding mottled pigmentation. Alternatively, the yellowish lesion may become smaller and hyperreflective photoreceptor bands transform into hyporeflective bands^(4)^. The hyperreflectivity may be associated with photoreceptor injury and disorganization. Moreover, hyporeflectivity may be related to photoreceptor apoptosis and atrophy. Similar to other laser-induced retinopathies, in our case, a focal, round, and yellowish lesion was detected in the fovea. Foveal outer retinal disruptions and irregularities extending from the external limiting membrane to the RPE were detected using OCT.

Currently, there is no consensus regarding the treatment of this pathology. Lim et al. reported the treatment of laser pointer maculopathy with tapering doses of oral prednisolone in a 13-year-old patient^(5)^. In addition, Turaka et al. treated a 13-year-old male patient with tapering doses of oral prednisolone (40 mg) for 3 weeks^(6)^.

Similarly, Combillet et al. used intravenous injections of high-dose corticosteroids for 3 days in a patient with laser-induced maculopathy and reported that retinal lesions decreased slightly^(7)^.

Anti-inflammatory and anti-proliferative effects of the steroids on RPE cells may recover the laser pointer-induced damage^(8)^. However, it is well established that steroid therapy is associated with both systemic and ocular side effects. Systemic side effects include osteoporosis, steroid-induced myopathy, increase in blood glucose levels, development of cushingoid features, impairment of growth in young children, and increased risk of infectious disease. Ocular side effects are cataract, glaucoma, and central serous chorioretinopathy.

Zhao et al. used oral lutein (20 mg) for 4 weeks in a patient with laser pointer-induced maculopathy. They observed improvement in visual acuity from 10/50 to 12/20 in 3 months^(9)^. At the end of the first year, the final visual acuity of the patient was 16/20. It is well established that lutein exerts anti-inflammatory and antioxidant effects, and has been used for macular disease, particularly age-related macular degeneration. However, due to the lack of a control case in that study, Zhao et al. stated that it was difficult to draw concrete conclusions regarding the effectiveness of treatment with lutein^(9)^.

On the other hand, some authors preferred to observe the patients. Tomasso et al. observed a 13-year-old boy with laser maculopathy; 1 month later, the BCVA had improved from 20/25 to 20/20, and the structural OCT showed complete resolution of the pathologic findings^(10)^. Similarly, Weng et al. showed that a patient with laser maculopathy demonstrated both visual and anatomic recovery during the follow-up period^(11)^.

In literature, most cases of laser-induced maculopathy are caused by laser pointer devices. In our case, laser-induced maculopathy was induced by a laser cutting machine. At the first visit, the ellipsoid zone damage and RPE damage was 215 µm and 534 µm, respectively. However, partial RPE damage was detected using OCT. In addition, there was no choriocapillaris damage. After 4 months, the ellipsoid zone damage had improved to 83 µm, and the RPE damage had almost complete resolved. The BCVA improved to 10/10. The choriocapillaris removes the waste products and provides nutrients to the RPE and neurosensory retina^(12)^. RPE cells have proliferative and migratory capacity^(10)^. An unaffected choriocapillaris may assist in the healing of RPE, and RPE cells may promote the healing of ellipsoid zone cells. Moreover, the present study revealed that visual and anatomical improvement can occur without any treatments in patients with laser-induced maculopathy.

In cases in which the damage is relatively small and the choriocapillaris is not affected, observation may be preferred. In this way, the possible side effects of steroid therapy can be prevented. In addition, the results of this study showed that the dominant eye is more susceptible to damage than the non-dominant eye. Wearing laser eye protection gear is very important for workers who use the laser cutting machine.

## References

[1] Barkana Y, Belkin M. (2000). Laser eye injuries. Surv Ophthalmol.

[2] Alsulaiman SM, Alrushood AA, Almasaud J, Alzaaidi S, Alzahrani Y, Arevalo JF, King Khaled Eye Specialist Hospital Collaborative Retina Study
Group (2014). High-power handheld blue laser-induced maculopathy: the results
of the King Khaled Eye Specialist Hospital Collaborative Retina Study
Group. Ophthalmology.

[3] King A, Gottlieb E, Brooks DG, Murphy MP, Dunaief JL. (2004). Mitochondria-derived reactive oxygen species mediate blue
light-induced death of retinal pigment epithelial cells. Photochem Photobiol.

[4] Hossein M, Bonyadi J, Soheilian R, Soheilian M, Peyman GA. (2011). SD-OCT features of laser pointer maculopathy before and after
systemic corticosteroid therapy. Ophthalmic Surg Lasers Imaging.

[5] Lim ME, Suelzer J, Moorthy RS, Vemuri G. (2014). Thermal macular injury from a 154 mW green laser
pointer. J AAPOS.

[6] Turaka K, Bryan JS, Gordon AJ, Reddy R, Kwong HM Jr, Sell CH (2012). Laser pointer induced macular damage: case report and mini
review. Int Ophthalmol.

[7] Combillet F, Saunier V, Rougier MB, Delyfer MN, Korobelnik JF. (2016). Multimodal imaging in a case of self-inflicted laser-induced
maculopathy. Eur J Ophthalmol.

[8] Ayalasomayajula SP, Ashton P, Kompella UB. (2009). Fluocinolone inhibits VEGF expression via glucocorticoid receptor
in human retinal pigment epithelial (ARPE-19) cells and TNF-alpha-induced
angiogenesis in chick chorioallantoic membrane (CAM). J Ocul Pharmacol Ther.

[9] Zhao N, Liu L. (2017). Long-term changes in optic coherence tomography in a child with
laser pointer maculopathy: A case report and mini review. Photodiagn Photodyn Ther.

[10] Tomasso L, Benatti L, La Spina C, Lattanzio R, Baldin G, Carnevali A (2017). Optical coherence tomography angiography findings in laser
maculopathy. Eur J Ophthalmol.

[11] Weng CY, Baumal CR, Albini TA, Berrocal AM. (2015). Self-induced laser maculopathy in an adolescent boy utilizing a
mirror. Ophthalmic Surg Lasers Imaging Retina.

[12] Booij JC, Baas DC, Beisekeeva J, Gorgels TG, Bergen AA. (2010). The dynamic nature of Bruch’s membrane. Prog Retin Eye Res.

